# Epigenetic effects of RRx-001: a possible unifying mechanism of anticancer activity

**DOI:** 10.18632/oncotarget.6526

**Published:** 2015-12-09

**Authors:** Hongjuan Zhao, Shoucheng Ning, Jan Scicinski, Bryan Oronsky, Susan J. Knox, Donna M. Peehl

**Affiliations:** ^1^ Department of Urology, Stanford University School of Medicine, Stanford, CA, USA; ^2^ Department of Radiation Oncology, Stanford University School of Medicine, Stanford, CA, USA; ^3^ EpicentRx, Inc., Mountain View, CA, USA

**Keywords:** RRx-001, epigenetics, cancer, DNA methylation, acetylation

## Abstract

RRx-001 is a novel aerospace-derived compound currently under investigation in several ongoing Phase II studies. In a Phase I trial, it demonstrated anti-cancer activity and evidence of resensitization to formerly effective therapies in heavily pre-treated patients with relapsed/refractory solid tumors. RRx-001 generates reactive oxygen and nitrogen species (ROS and RNS) and nitric oxide (NO), elicits changes in intracellular redox status, modulates tumor blood flow, hypoxia and vascular function and triggers apoptosis in cancer cells. We investigated the effect of RRx-001 on the epigenome of SCC VII cancer cells. RRx-001 at 0.5 and 2 μM significantly decreased global DNA methylation, i.e., 5-methylcytosine levels, in SCC VII cells. Consistently, 0.5-5 μM RRx-001 significantly decreased Dnmt1 and Dnmt3a protein expression in a dose- and time-dependent manner. In addition, global methylation profiling identified differentially methylated genes in SCC VII cells treated with 0.5, 2, and 5 μM RRx-001 compared to control cells. Twenty-three target sites were hypomethylated and 22 hypermethylated by >10% in the presence of at least two different concentrations of RRx-001. Moreover, RRx-001 at 2 μM significantly increased global acetylated histone H3 and H4 levels in SCC VII cells after 24 hour treatment, suggesting that RRx-001 regulates global acetylation in cancer cells. These results demonstrate that, in contrast to the traditional “one drug one target” paradigm, RRx-001 has multi(epi)target features, which contribute to its anti-cancer activity and may rationalize the resensitization to previously effective therapies observed in clinical trials and serve as a unifying mechanism for its anticancer activity.

## INTRODUCTION

RRx-001 (also known as ABDNAZ, 1-bromoacetyl-3,3-dinitroazetidine) is a novel aerospace-derived compound under active investigation as a chemo-, immuno- and radiosensitizer in Phase II clinical trials. In Phase I, it demonstrated encouraging evidence of antitumor activity including resensitization to formerly effective chemotherapy while exhibiting a benign safety profile in heavily pre-treated patients with relapsed/refractory solid tumors [1]. With a pharmacologically unprecedented per-nitro heterocyclic scaffold, RRx-001 has also shown promising activity as a radiosensitizer [2, 3] in addition to activity in a variety of important pathological conditions besides cancer including malaria [4], hemorrhagic shock [5] and sickle cell anemia [6]. The multiple mechanisms of activity in cancer include generation of reactive oxygen and nitrogen species (RONS), including nitric oxide (NO), modulation of intracellular redox status, modulation of tumor-selective vascular blood flow and induction of apoptosis [3]. Additional mechanisms remain to be elucidated.

The ubiquitous nature of epigenetic alterations in all types of cancer underlies their important oncogenic role [7–9]. For example, abnormal DNA methylation is a feature of most cancers, and mutations in the enzymes that add methyl groups to DNA have been associated with some cancer types. The inherent malleability and adaptability of the epigenome, in contrast to the relative stability of the genome, suggests the possibility of therapeutic intervention; indeed, epigenetic therapy has emerged as a novel treatment strategy to sensitize or “prime” solid tumors (especially ovarian cancer), leukemias and lymphomas to standard chemotherapy, immunotherapy and radiation [10]. Two inhibitors of DNA methyltransferases, azacytidine (vidaza) and 5-aza-2′-deoxycytidine (decitibine), have already been approved by the Food and Drug Administration (FDA) as effective drugs for treatment of patients with myelodysplastic syndromes [11]. An inhibitor of histone deacetylases, vorinostat (suberoylanilide hydroxamic acid), is approved for the treatment of cutaneous T-cell lymphoma [12]. Other epigenetic drugs targeting histone modifying enzymes or DNA methylation are in clinical trials or development [7].

Here, these experiments determined the effects of RRx-001 on the epigenome of cancer cells. Squamous cell carcinoma (SCC VII) cells were treated with RRx-001 at its therapeutic concentrations (0.5-5 μM) and global DNA methylation, i.e., 5-methylcytosine levels, was determined by ELISA. In addition, global methylation profiling of treated and control cells was carried out using Illumina Infinium HumanMethylation450 BeadChips^®^. Expression of Dnmt1 and Dnmt3a proteins was examined in treated and control cells by Western blot. Moreover, global levels of acetylated histone H3 and H4 levels in SCC VII cells were determined by a fluorometric assay. These results, which demonstrate modification of both the DNA methylation and histone acetylation status in cancer cells, characterize RRx-001 as a pan-epigenetic inhibitor.

## RESULTS

### RRx-001 significantly decreased global 5-methylcytosine (5-mC) levels

**Figure 1:**
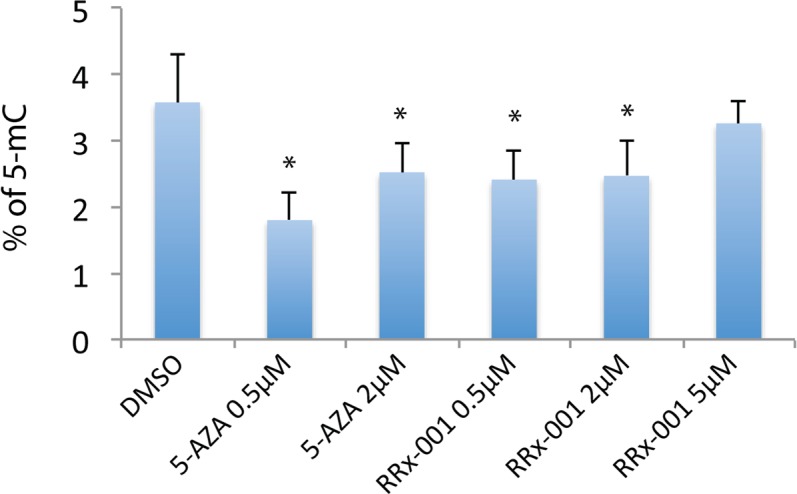
Determination of the effect of RRx-001 on the level of global 5-mC by ELISA Genomic DNA from SCC VII cells treated with DMSO (0.025%), 0.5 uM or 2 uM 5-azacytidine (5-AZA), and 0.5 uM, 2 uM, or 5 uM RRx-001 for 72 hours was used to determine the 5-mC level in an ELISA assay. * indicates statistical significance determined by Student's *t*-test.

To determine the effect of RRx-001 on global 5-mC levels in SCC VII cells, the cells were treated with 0.5, 2 or 5 μM RRx-001 for 72 hours and 5-mC levels were measured by ELISA. For comparison, cells were also treated with 5-azacytidine, a known epigenetic modifier causing DNA demethylation. As expected, 5-azacytidine at 0.5 and 2 μM significantly decreased global 5-mC levels in SCC VII cells (Figure 1). Treatment of SCC VII cells with 0.5 and 2 μM RRx-001 also significantly decreased global 5-mC levels (Figure 1), demonstrating demethylating capability of RRx-001. Of note, the degree of demethylation induced by 5-azacytidine was greater at 0.5 μM than at 2 μM, consistent with previous findings that, in some cases, lower concentrations of 5-azacytidine elicited a greater effect on methylation than higher concentrations, when DNA damage and apoptosis become more prominent [13, 14]. Interestingly, RRx-001 also induced epigenetic effects at lower rather than higher concentrations: whereas 0.5 and 2 μM demonstrated equivalent demethylating potency, 5 μM did not significantly alter 5-mC levels from untreated controls. These results indicate that RRx-001 is a demethylating agent in cancer cells at therapeutic concentrations of 0.5 - 2 μM.

### RRx-001 altered methylation of genes involved in pathways relevant to cancer

**Figure 2:**
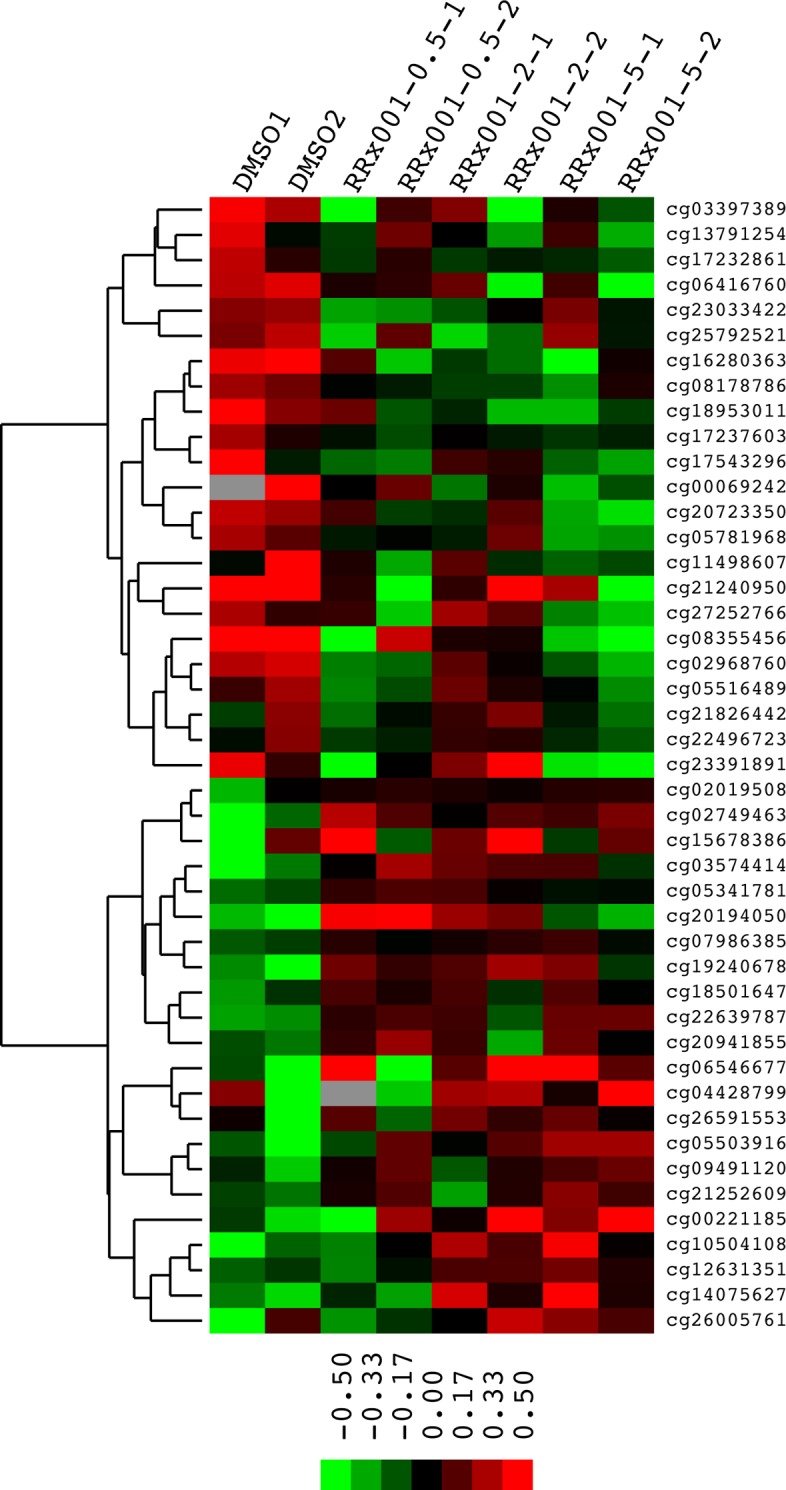
Heat map of methylation levels of CpG sites upregulated or downregulated by >10% by at least 2 concentrations of RRx-001 Each row represents a CpG site and the name of the site is given at the right side of the image. Each column represents a treatment condition noted on top of each column. Sites are clustered based on their patterns of change after treatment. Data were log2 transformed and centered. The scale bar shows the direction and the distance of the methylation level from the median.

To determine gene-specific effects of RRx-001 on DNA methylation on a genome-wide scale, DNA methylation profiles of SCC VII cells treated with either DMSO as a control and 0.5, 2 or 5 μM RRx-001 were analyzed using Illumina Infinium HumanMethylation450 BeadChips^®^. The utility of Illumina Infinium HumanMethylation450 BeadChips^®^ in interrogating mouse DNA methylation profiles has been demonstrated recently by Wong et al. [15]. Of the 13,715 probe sequences matched to bisulfite-converted mouse genomic DNA, 12,023 had a detection p-value <0.05 across all samples. RRx-001 decreased methylation levels of 5 target sites by >10% across all concentrations compared to control (Table 1). In addition, 23 target sites were demethylated by >10% in at least two treatments (Figure 2). Similarly, RRx-001 increased methylation levels of 2 target sites by >10% across all concentrations compared to control (Table 1) and 22 target sites were hypermethylated by >10% in at least two treatments (Figure 2). These results provide direct evidence that RRx-001 consistently regulated DNA methylation in SCC VII cells.

**Table 1:** Target sites with >10% change of methylation across all concentrations

			% change of methylation		
TargetID	Gene symbol	Position	0.5 μM	2 μM	5 μM
cg11498607	RUNX1	Body	−16.73	−10.10	−18.29
cg20723350	PPARGC1A	1stExon	−11.48	−10.26	−22.17
cg03397389	EIF2C3	1stExon	−12.42	−10.81	−10.05
cg17237603	ZNF398	Body	−13.86	−10.92	−13.55
cg16280363	MARS	Body	−11.12	−12.21	−13.01
cg03574414	CHRNA2	Body	14.70	14.83	10.25
cg02019508	ATF7	5′UTR	12.47	11.04	13.14

**Figure 3:**
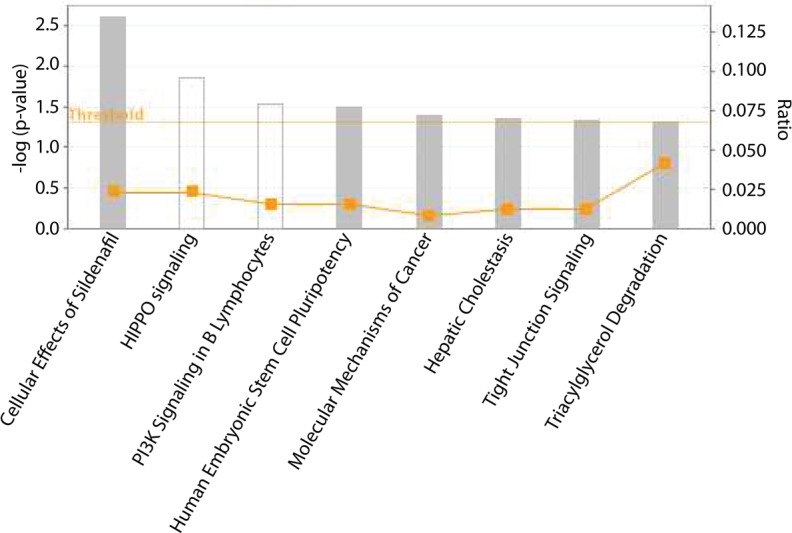
Pathways identified by IPA that are enriched in the list of genes whose methylation levels are affected by RRx-001 Threshold indicates statistical significance of *p* < 0.05 determined by IPA. Square indicates the ratio of overlapping genes between gene list of interest and pathways annotated in IPA.

To determine the gene pathways affected by changes in methylation induced by RRx-001, pathway enrichment analyses were performed using IPA. Eight pathways were significantly enriched in the 23 hypomethylated and 22 hypermethylated genes (Figure 3), including PI3K signaling in B lymphocytes, human embryonic stem cell pluripotency and molecular mechanisms of cancer.

### RRx-001 inhibited expression of Dnmt1 and Dnmt3a in SCC VII and PANC-1 cells

**Figure 4:**
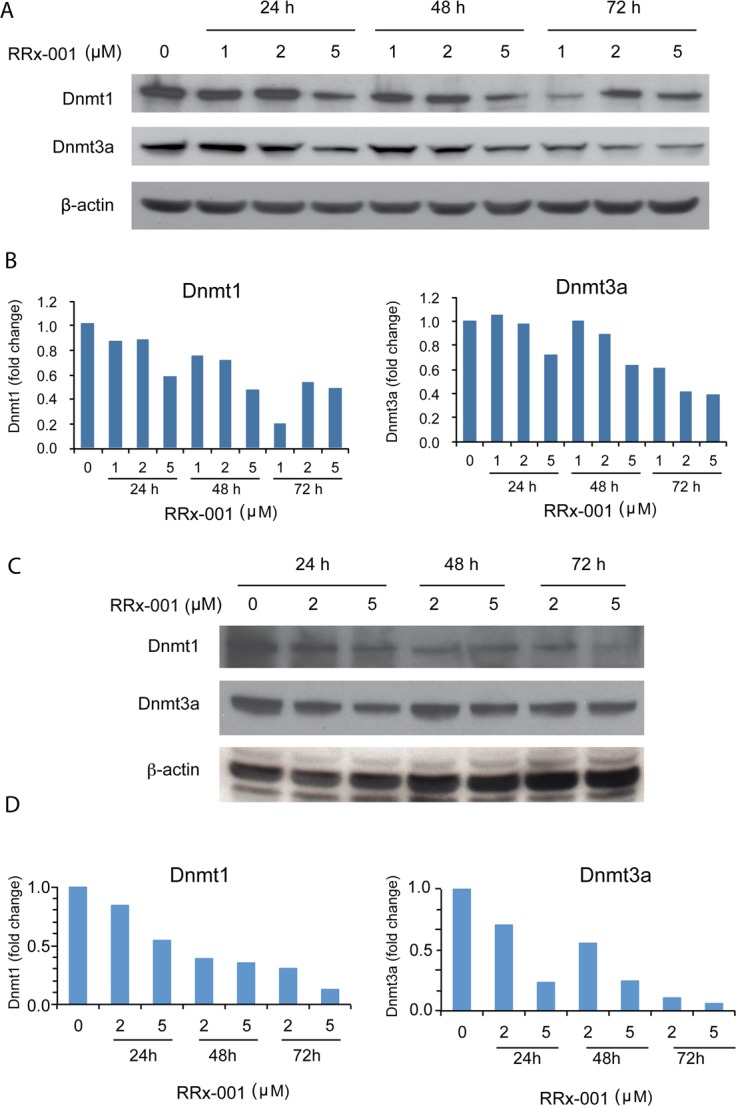
Determination of the effect of RRx-001 on the protein levels of Dnmt1 and Dnmt3a by western blot Whole cell lysates were prepared from cells treated with diluent or 1-5 μM RRx-001 for 1-3 days. **A.** and **C.** Images of blots for SCC VII and PANC-1 cells, respectively. **B.** and **D.** Relative density of Dnmt1 and Dnmt3a bands determined by Image J, normalized to β-actin for SCC VII and PANC-1 cells, respectively.

To investigate the mechanism by which RRx-001 decreased global 5-mC levels, the protein levels of two DNA methyltransferases, Dnmt1 and Dnmt3a, were evaluated by Western blot. As shown in Figure 4A and 4B, the levels of Dnmt1 and Dnmt3a proteins in SCC VII cells were reduced gradually over the 3-day exposure to 1-5 μM RRx-001 in a dose-dependent manner. Daily refreshment of RRx-001 resulted in greater inhibition of Dnmt1 and Dnmt3a expression in SCC VII cells compared to single-administration RRx-001 (not shown). In addition, RRx-001 decreased the levels of Dnmt1 and Dnmt3a proteins in human PANC-1 cells in a dose-dependent manner (Figure 4C and 4D). These results demonstrated that RRx-001 suppressed Dnmt1 and Dnmt3a protein expression in both murine and human cancer cells, suggesting that RRx-001 decreased global DNA methylation in SCC VII cells by modulating levels of these proteins.

### RRx-001 significantly increased global acetylated histone H3 and H4 levels

**Figure 5:**
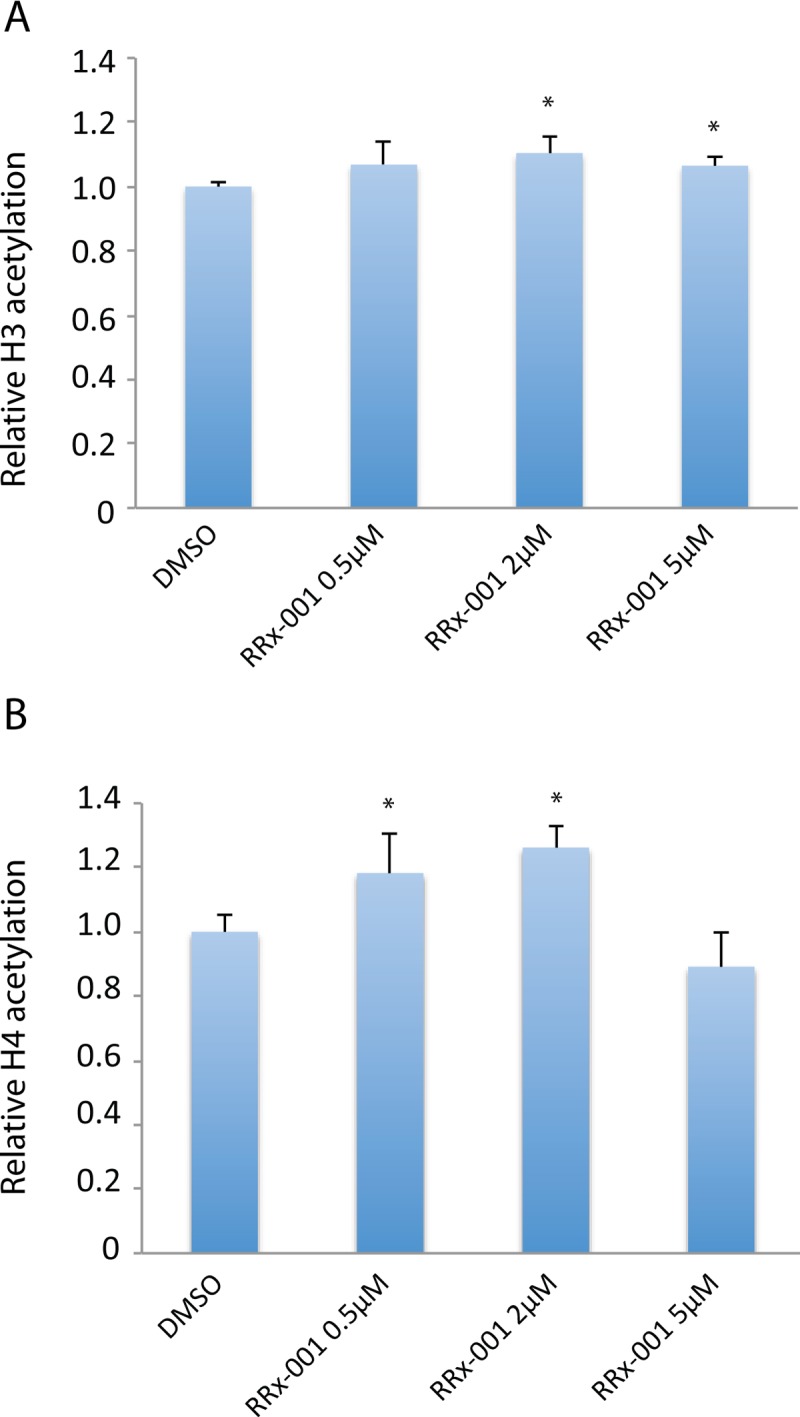
Determination of the effect of RRx-001 on the levels of acetylated H3 and H4 proteins Total histone extracts were prepared from SCC VII cells after 24 hours of treatment with either DMSO or 0.5-5 μM RRx-001. Levels of acetylated histone H3 and histone H4 were determined using a fluorometric assay. * indicates statistical significance determined by Student's *t*-test.

To evaluate effects of RRx-001 on histone acetylation, a fluorometric assay was used to measure total acetylated histone H3 and H4 levels in SCC VII cells after 24 hours of treatment with the drug. As shown in Figure 5, RRx-001 significantly increased the level of total acetylated histone H3 protein at 2 μM and 5 μM compared to control. In addition, the level of total acetylated histone H4 protein was significantly increased by 0.5 μM and 2 μM RRx-001, showing that RRx-001 upregulates global acetylation in SCC VII cells.

## DISCUSSION

Emerging data from ongoing clinical trials with RRx-001 implicate epigenetic modification of gene expression as a major mechanism by which it elicits both single-agent antitumor activity and sensitization/resensitization to standard chemotherapies [16]. Results from a Phase I trial in heavily pretreated patients with advanced/refractory solid tumors were notable for the lack of systemic toxicity at all doses tested and evidence of anticancer efficacy, with 15 of 25 patients exhibiting partial response or stable disease [1]. Furthermore, after progression on RRx-001 treatment, 4 patients were rechallenged with drugs to which they had previously acquired resistance, and all 4 exhibited resensitization [1]. Preliminary results from ongoing Phase II clinical trials have recapitulated the favorable safety and tolerability profile from Phase I and provided initial confirmation of antitumor priming to subsequent therapy post-RRx-001. To date, in a randomized Phase II trial against regorafenib, 9 of 12 irinotecan-refractory RRx-001 colorectal patients have been resensitized to irinotecan, which contrasts sharply with the experience of regorafenib patients who have been too debilitated and systemically unwell to receive any subsequent salvage therapies [17]. Since reversal of epigenetic-induced gene silencing has been linked to chemo-resensitization [10, 18], these experiments were designed to investigate whether RRx-001 affects the epigenome. In the syngeneic SCC VII preclinical model of squamous cell carcinoma that has been used extensively to study the activity of RRx-001 [2, 19], RRx-001 decreased both global DNA 5-mC levels and increased histone H3 and H4 acetylation at clinically relevant therapeutic doses. These activities are relevant to anti-cancer efficacy of RRx-001 as a single agent as well as to its potential as a chemo-, radio- and immunosensitizer.

DNA methylation is an epigenetic event that regulates chromatin compaction and gene expression, and silencing of tumor suppressor genes by hypermethylation is a common feature of cancer [19]. Enzymes that catalyze the transfer of methyl groups onto cytosine residues are Dnmt1, Dnmt3a and Dnmt3b [19]. Dnmt1 is a “copy” DNA methyltransferase that maintains methylation once epigenetic marks are in place. Dnmt3a and Dnmt3b are *de novo* methyltransferases, which establish the initial patterns of DNA methylation. DNA methylation inhibitors in clinical use (decitabine and vidaza) and other similar nucleoside analogues incorporate into DNA during replication and block further DNA methylation. Decitabine inhibits Dnmt by forming an irreversible covalent bond with the active site of the enzyme, depletes Dnmt through proteosomal degradation, and induces global DNA hypomethylation [20, 21]. Like the nucleoside analogues, RRx-001 reduced protein levels of Dnmt1 and Dnmt3a in a time- and dose-dependent manner and decreased global DNA methylation. Moreover, RRx-001, which does not fit the standard profile of a molecularly targeted agent, is likely to interfere indirectly with the DNA methylation pathway rather than directly like decitabine and vidaza. Oxidative stress has been linked to loss of methylation due to ROS-induced DNA damage. The DNA lesions caused by ROS can impede the ability of DNA to function as a substrate for Dnmts, resulting in global hypomethylation [22, 23]. In addition, excess oxidative stress, which decreases glutathione levels, also leads to undermethylation, possibly through depletion of the primary methyl group donor, S-adenosylmethionine [24]. Furthermore, H_2_O_2_ may inactivate the DNA methyltransferases by oxidation of the catalytic-site cysteine residue in the active site [25]. Since RRx-001 propagates runaway ROS in tumors [3], its inhibitory effects on methylation and Dnmt activity may be at least partly due to the excess accumulation of free radicals.

In this study, RRx-001 also increased acetylation of histones H3 and H4, suggesting that RRx-001 is an inhibitor of histone deacetylases (HDACs). Histone deacetylase inhibitors (HDACi) are compounds that block the activity of Zn^++^-dependent HDACs, inducing hyperacetylation of histones and other proteins [26]. Histone acetylation is associated with open chromatin structure, and it is believed that the anti-cancer effects of HDACi are in part due to the reactivation of suppressor genes. HDACs are also linked to the initiation and maintenance of repression of DNA hypermethylated genes. Romidepsin and vorinostat are two FDA-approved HDACi for hematologic malignancies [27]. Since oxidative stress has been linked to the inhibition of HDAC activity, which promotes the acetylation of histones, it follows that the RRx-001-induced hyperacetylation of histones H3 and H4 may be associated with ROS formation [28].

Despite minimal activity as single agents, HDACi have been shown to reverse chemoresistance [29]. Similarly, azacitidine sensitizes advanced cancers to platinum therapy [30]. Decitabine is a forerunner in epigenetic therapy and may overcome resistance to molecular targeted therapy, induce cell reprogramming, and elicit immune responses through epigenetic modifications [20]. However, in a recent Phase II clinical trial, decitabine reduced rather than increased efficacy of carboplatin in partially platinum-sensitive ovarian cancer [31]. Overall, these clinical proof-of-concept studies to establish epigenetic therapies as multidrug resistant-reversal agents have lacked consistency [10, 18].

Combination therapy with methyltransferase inhibitors and HDACi may prove more efficacious than treatment with either class of drug alone [32, 33]. For example, azacytidine in combination with the HDACi, entinostat, demonstrated evidence of efficacy in patients with refractory non-small cell lung cancer, as well as subsequent resensitization to previously refractory drugs [34]. As a multi-targeted systemically non-toxic anticancer agent with broad-spectrum activity on DNA methylation and histone acetylation, RRx-001 has the properties of an “all-in-one combination” epi-therapy without any of the adverse effects of Dnmt or HDAC inhibitors. These present findings add new facets to the known anticancer activity of RRx-001.

The downstream action of RRx-001 on tumors first involves an initial binding event to the beta-93 cysteine residue of hemoglobin to form adducts in the red blood cell [35, 36]. The conformational changes induced in hemoglobin lead to an increase in oxidative stress within the red blood cell, followed by deposition of denatured hemoglobin on the membrane and other membrane effects due to oxidative stress. Covalent binding of RRx-001 to hemoglobin also increases the rate of nitrite reduction to NO under hypoxia [37]. As the intermediaries of cytotoxicity, RRx-001-modified red blood cells occlude tumor microvasculature, are internalized by tumor endothelial cells, and release heme, iron and lipids upon catabolism inside the tumor. These redox-active heme degradation products, membrane-free iron and lipids derived from the RBC membrane mediate oxidative damage in the tumor microenvironment. There is also evidence that RRx-001 causes vascular normalization and increases blood flow in tumors [38].

The results of our current study add epigenetic modulation to the list of anti-cancer properties of RRx-001, likely through activity on DNA methyltransferases and histone deacetylases that contain redox-sensitive active site cysteines. Similar to the diverse activity of the DNA hypomethylators and HDAC inhibitors, which may profoundly affect gene expression patterns in cancer, RRx-001 induces pleiotropic antitumor effects. Taken together, these results demonstrate that, in contrast to the traditional “one drug one target” paradigm, RRx-001 has multi(epi)target features that contribute to its anti-cancer activity and may represent a single unifying mechanism accounting for its broad range of antitumor effects.

## MATERIALS AND METHODS

### Reagents

RRx-001 was obtained from Orbital ATK. The DNA methyltransferase inhibitor 5-azacytidine was purchased from Sigma-Aldrich. RRx-001 and 5-azacytidine solutions were freshly prepared on the experimental day by dissolving in DMSO and then diluting with cell culture medium. The final concentration of DMSO was 0.025%. Antibodies against Dnmt1, Dnmt3a, Δ-actin and HRP-conjugated secondary antibodies were purchased from Santa Cruz Biotechnology.

### Cell treatment

Murine SCC VII and human PANC-1 cells were grown in DMEM medium supplemented with 10% fetal calf serum, 100 units/ml penicillin, and 100 μg/ml streptomycin in a 37°C humidified incubator with a mixture of 95% air and 5% CO2. Exponentially growing cells were plated in 100-mm dishes with 5-10 × 10^5^ cells per dish in DMEM culture media and grown overnight. Cells were treated with RRx-001, 5-azacytidine or 0.025% DMSO as a vehicle control for one to three days. Media containing RRx-001, 5-azacytidine, or DMSO were replaced daily over the 3-day period.

### Global DNA methylation profiling

Genomic DNA was isolated using a Qiagen AllPrep DNA/RNA Mini Kit (Qiagen) from SCC VII cells treated with the following compounds for 72 hours: DMSO (0.025%); 0.5 μM or 2 μM 5-azacytidine; and 0.5 μM, 2 μM, or 5 μM RRx-001. One μg of genomic DNA from each treated specimen was submitted to the Stanford Functional Genomics Facility. Global methylation profiling was performed using Illumina Infinium HumanMethylation450 BeadChips^®^ (Illumina) according to the manufacture's instructions. Raw data was deposited into GEO (accession number GSE74797). The methylation levels of genes that changed at least 10% were identified in Excel. Pathways affected by RRx-001 were identified using Ingenuity Pathway Analysis (IPA) (Qiagen).

### Quantitating 5-methylcytosine (5-mC) levels

Genomic DNA (100 ng) was isolated as described above from SCC VII cells treated with DMSO (0.025%); 0.5 μM or 2 μM 5-azacytidine; and 0.5 μM, 2 μM, or 5 μM RRx-001 for 72 hours. 5-mC levels were determined using a 5-mC DNA ELISA Kit (Zymo Research) according to the manufacture's instructions. Student's t-test was used to determine statistical significance in Excel.

### Quantitating total H3 or H4 acetylation levels

Histone protein extract (1-2 μg) was isolated from SCC VII cells treated with DMSO (0.025%), 0.5 μM, 2 μM, or 5 μM RRx-001 for 24 hours. Total H3 or H4 acetylation levels were determined using Histone H3 or Histone H4 Total Acetylation Detection Fast Kits (Abcam) according to the manufacture's instructions. Student's t-test was used to determine statistical significance in Excel.

### Western blots

After treatment with diluent or 1-5 μM RRx-001 for 1-3 days, cells were washed twice with cold phosphate-buffered saline and lysed in RIPA buffer (Sigma) for whole cell lysates. The protein contents were quantified using a Bio-Rad Quick Start protein assay kit (Hercules). Samples containing equal amounts of total protein (20 ug) were separated on 10% SDS-PAGE gels and transferred onto PVDF membranes. The membranes were blocked with 5% non-fat milk and probed with primary antibodies against Dnmt1 (1:500), Dnmt3a (1:500) and Δ-actin (1:500), and HRP-conjugated secondary antibodies (1:2500). The immunoreactive proteins were detected with ECL plus chemiluminescence detection reagents (Amersham Biosciences). The Western blot analyses were run at least twice for each molecular marker, unless otherwise indicated.
